# Ghrelin Facilitates GLUT2-, SGLT1- and SGLT2-mediated Intestinal Glucose Transport in Goldfish (*Carassius auratus*)

**DOI:** 10.1038/srep45024

**Published:** 2017-03-24

**Authors:** Ayelén Melisa Blanco, Juan Ignacio Bertucci, Naresh Ramesh, María Jesús Delgado, Ana Isabel Valenciano, Suraj Unniappan

**Affiliations:** 1 Departamento de Fisiología (Fisiología Animal II), Facultad de Biología, Universidad Complutense de Madrid, Madrid, Spain; 2 Laboratory of Integrative Neuroendocrinology, Department of Veterinary Biomedical Sciences, Western College of Veterinary Medicine, University of Saskatchewan, Saskatoon, Saskatchewan, Canada; 3 Instituto de Investigaciones Biotecnológicas-Instituto Tecnológico Chascomús, Buenos Aires, Argentina

## Abstract

Glucose homeostasis is an important biological process that involves a variety of regulatory mechanisms. This study aimed to determine whether ghrelin, a multifunctional gut-brain hormone, modulates intestinal glucose transport in goldfish (*Carassius auratus*). Three intestinal glucose transporters, the facilitative glucose transporter 2 (GLUT2), and the sodium/glucose co-transporters 1 (SGLT1) and 2 (SGLT2), were studied. Immunostaining of intestinal sections found colocalization of ghrelin and GLUT2 and SGLT2 in mucosal cells. Some cells containing GLUT2, SGLT1 and SGLT2 coexpressed the ghrelin/growth hormone secretagogue receptor 1a (GHS-R1a). Intraperitoneal glucose administration led to a significant increase in serum ghrelin levels, as well as an upregulation of intestinal *preproghrelin, ghrelin O-acyltransferase* and *ghs-r1* expression. *In vivo* and *in vitro* ghrelin treatment caused a concentration- and time-dependent modulation (mainly stimulatory) of GLUT2, SGLT1 and SGLT2. These effects were abolished by the GHS-R1a antagonist [D-Lys3]-GHRP-6 and the phospholipase C inhibitor U73122, suggesting that ghrelin actions on glucose transporters are mediated by GHS-R1a *via* the PLC/PKC signaling pathway. Finally, ghrelin stimulated the translocation of GLUT2 into the plasma membrane of goldfish primary intestinal cells. Overall, data reported here indicate an important role for ghrelin in the modulation of glucoregulatory machinery and glucose homeostasis in fish.

Glucose is a critical source of energy for most physiological processes, especially the functioning of brain. Circulating glucose comes mainly from the diet during the fed state, and from gluconeogenesis and glycogenolysis during fasting^1^. Diet-derived glucose is absorbed by enterocytes through specific glucose transporters or carriers. These transporters are classified into two families: facilitative glucose carriers (GLUTs), through which glucose is transported by facilitated diffusion, and sodium/glucose co-transporters (SGLTs), which co‐transport Na^+^ and glucose by an electrochemical gradient across the membrane^2^. The classical model of glucose absorption through the intestine in mammals indicates that SGLT1 transports glucose from the intestinal lumen to the cytosol of enterocytes^3^, whereas GLUT2 is in charge of transporting glucose from cytosol to the blood^3^,^4^. Additionally, after high luminal glucose, GLUT2 is usually translocated from intracellular vesicles into the apical membrane, allowing bulk absorption of glucose^5^,^6^. GLUT2 and SGLT1 have been also proposed to function as glucose sensors in the intestine and other tissues of mammals^3^. Apart from these two transporters, the presence of small quantities of SGLT2 was detected in the rat intestine^7^, suggesting that this transporter (primarily involved in glucose uptake in the kidney) might also participate in glucose transport across the intestinal walls.

In fish, the mechanisms of carbohydrate absorption are believed to be very similar to those described in mammals. Thus, the entry of glucose across the brush-border membrane of the fish intestinal cells is SGLT1-driven^8^,^9^,^10^, whereas a Na+ -independent, facilitated carrier mediates glucose transport across the basolateral membrane to the blood^9^,^11^,^12^. The presence of GLUT2 in the intestine of several fishes^13^,^14^ suggests that this member of the GLUT family is such a facilitated carrier, although the precise location of this transporter within the enterocytes is yet to be investigated. As in mammals, both GLUT2 and SGLT1 were demonstrated to have glucosensory properties in fish^15^,^16^. Fish exhibit a wide range of natural feeding habits and consume herbivorous, carnivorous and omnivorous diets. These diverse feed composition determine different capabilities of the intestinal uptake process, and hence different glucose absorption rates depending on the species^9^,^17^,^18^,^19^.

Intestinal glucose absorption, and glucose metabolism in general, is subjected to a strict endocrine control that acts to keep blood glucose levels in a narrow range. Classical glucoregulatory hormones include the pancreas-derived insulin and glucagon, and the intestinal glucagon-like peptide 1 (GLP-1) and glucose-dependent insulinotropic peptide (GIP)^20^,^21^. However, other newer hormones have been involved in the regulation of glucose homeostasis. One of them is ghrelin, a peptide hormone mainly synthesized by the stomach and/or the foregut^22^,^23^. Ghrelin is particularly important for being the only peripheral protein known to be posttranslationally modified by an acylation. This modification that is essential for ghrelin activity is catalysed by ghrelin O-acyl transferase (GOAT)^24^,^25^. Besides, it is the only peripheral peptide with a stimulatory (orexigenic) role in food intake in both mammals^26^ and fish^27^.

The role of ghrelin in glucose metabolism, in mammals, is mainly associated with an elevation in blood glucose levels due to a decrease in insulin secretion, thus exerting an insulinostatic action^28^,^29^. In terms of glucose transport, ghrelin has been reported to reduce both GLUT2 levels and the glucose uptake in primary astrocytes from rat hypothalamus^30^, to increase insulin-induced glucose uptake in isolated rat white adipocytes^31^, and to enhance *GLUT4* mRNA expression in rat cultured myocytes^32^. In fish, the involvement of ghrelin in the regulation of glucose metabolism has been less explored, but it is suggested that this peptide regulates carbohydrate metabolism via insulin inhibition and glucagon stimulation in zebrafish^33^. Additionally, ghrelin was reported to increase GLUT2 levels in rainbow trout hypothalamus and hindbrain^34^. Overall, there are few studies in mammals and fish showing a connection between ghrelin and glucose transport regulation in locations different to the intestine, but no reports are available on ghrelin and intestinal glucose transport.

Ghrelin actions are mediated by G-protein coupled receptors known as growth hormone secretagogue receptors (GHS-R), or ghrelin receptors^22^,^35^. Different subtypes of ghrelin receptors have been described in mammals and fish^36^,^37^, among which GHS-R1a seems to be the one mediating most physiological functions of ghrelin^37^,^38^,^39^. This receptor predominantly triggers the phospholipase C (PLC)/protein kinase C (PKC) and/or the adenylyl cyclase (AC)/protein kinase A (PKA) intracellular pathways for signal transduction^39^,^40^,^41^. Nevertheless, other molecular pathways, including the AMP-activated protein kinase (AMPK)^42^, the mitogen-activated protein kinase (MAPK)^43^,^44^ and the mechanistic target of rapamycin (mTOR)^45^ pathways, have been also associated to actions mediated by GHS-R1a.

In the present study, we aimed to characterize possible interactions between the ghrelinergic system and the machinery involved in glucose absorption in fish, using goldfish (*Carassius auratus*) as the animal model. Very recently we found that ghrelin is likely helping carbohydrate digestion by increasing the glucosidase enzyme sucrase-isomaltase (Blanco *et al*., *under review*). Here we evaluated whether ghrelin modulates intestinal glucose transport, thus trying to elucidate a possible role for this hormone in carbohydrate absorption. It is particularly important to better understand the mechanisms underlying glucose metabolism and utilization in fish, as many species are known to show a poor ability to utilize dietary carbohydrates^46^. Given this importance, numerous studies have been published characterizing glucose metabolism and glucose sensing in fishes^9^,^16^. To achieve our purpose, we first characterized the cellular localization of glucose transporters GLUT2, SGLT1 and SGLT2 in relation to ghrelin, GOAT and GHS-R1a within the goldfish intestine. Then, we studied whether the ghrelinergic system is modulated by glucose, by characterizing the expression of mRNAs encoding ghrelin, GHS-R1a and GOAT after glucose administration. Does ghrelin affect glucose transporters in goldfish intestine? For this we used a double approximation, testing the effects of ghrelin treatment on the intestinal expression (mRNA and/or protein) of GLUT2, SGLT1 and SGLT2 both *in vivo* and *in vitro*. The results obtained indicate that ghrelin, in a time- and concentration-dependent manner, stimulates the expression of GLUT2, SGLT1 and SGLT2. We then investigated whether GHS-R1a is mediating such effects, and the possible implication of the PLC/PKC and AC/PKA intracellular signal transduction pathways. Finally, using a primary culture of goldfish intestinal cells, we studied if the effects of ghrelin on intestinal glucose transporters include a modulation of their translocation rate into the plasma membrane. The information gained is of great importance for better understanding the physiological actions of the ghrelinergic and glucoregulatory systems in fish.

## Results

### The glucose transporters GLUT2, SGLT1 and SGLT2 are present in the goldfish intestine

**Figure 1:**
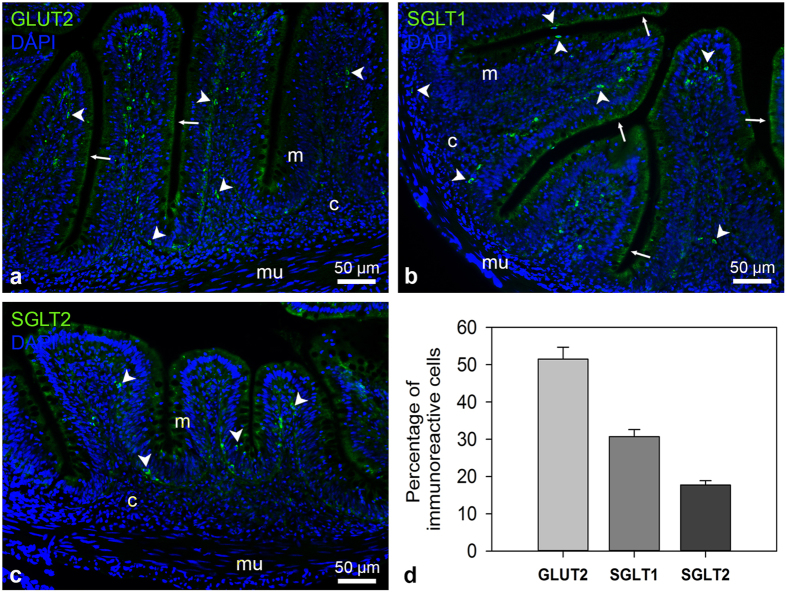
GLUT2-like, SGLT1-like and SGLT2-like immunoreactivity in goldfish intestine detected by immunohistochemistry. (**a–c**) Representative transverse sections of intestine showing GLUT2-like (a; green), SGLT1-like (b; green) or SGLT2-like (**c**; green) immunoreactivity. All images are merged with DAPI showing nuclei in blue. Arrowheads (➤) indicate immunoreactive cells and solid thin arrows (→) point glucose transporter labeling in the apical membrane. Scale bars (μm) are indicated in each image. c, connective tissue (lamina propria + submucosa); m, mucosa; mu, muscular layer. Images were captured using a Nikon Eclipse Ti-Inverted fluorescence microscope. **(d)** Relative abundance of GLUT2, SGLT1 and SGLT2 positive cells in the intestine of goldfish. Data are presented as media percentage + SEM of each population of cells in three different sections. For details on methods employed for percentage calculation, please consult the *Methods* section.

**Figure 2:**
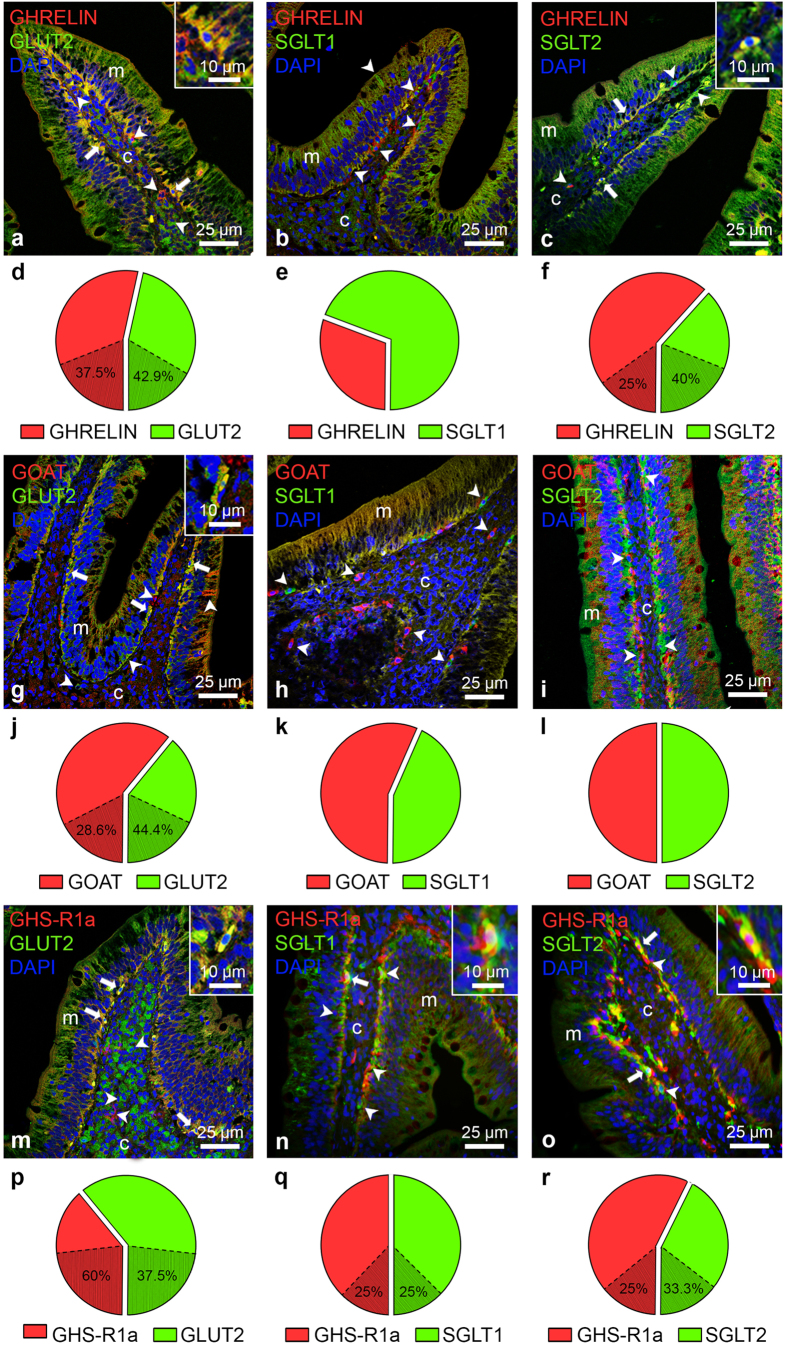
Ghrelin-like/GOAT-like/GHS-R1a and GLUT2-like/SGLT1-like/SGLT2-like double immunostaining in goldfish intestine assessed by immunohistochemistry. (**a–c, g–i, m–o**) Representative transverse sections of intestine showing merged images of ghrelin (red) and GLUT2, SGLT1 and SGLT2 (green) (**a–c**), GOAT (red) and GLUT2, SGLT1 and SGLT2 (green) (**g–i**) and GHS-R1a (red) and GLUT2, SGLT1 and SGLT2 (green) (**m–o**). Cells that show colocalization between the two assessed molecules are stained in yellow. All images are merged with DAPI showing nuclei in blue. Arrowheads (➤) indicate cells stained with either ghrelin/GOAT/GHSR-1a or GLUT2/SGLT1/SGLT2, and solid arrows (→) show cells that colocalize both ghrelin/GOAT/GHS-R1a and each of the glucose transporters. A magnified image of representative cells positive to both ghrelin/GOAT/GHSR-1a and GLUT2/SGLT1/SGLT2 is shown in square inset in each figure. Scale bars (μm) are indicated in each image. (**c)** connective tissue (lamina propria + submucosa); (**m**), mucosa. Images were captured using a Leica TCS SP5 confocal microscope, except for n and o which were captured using a fluorescence microscope. (**d–f, j–l, p–r**) Relative abundance of ghrelin/GOAT/GHS-R1a (red) and GLUT2/SGLT1/SGLT2 (green) cells in the intestine of goldfish. Data are presented as relative abundance of each of the two cell-types studied in each case, assessed by counting the total number of the different cells in three sections. The percentage of each cell-type that colocalizes with the other is shadowed and indicated in the figure.

Immunostaining of goldfish intestinal sections revealed the presence of round or elongated GLUT2-like (green; Figs 1a and 2a,g,m, Supplementary Figs S1–S3), SGLT1-like (green; Figs 1b and 2b,h,n, Supplementary Figs S1–S3) and SGLT2-like (green; Figs 1c and 2c,i,o, Supplementary Figs S1–S3) cells in the villi. The great majority of the cells immunopositive for each of the transporters was observed along the border of the mucosa, adjacent to the lamina propria. A few GLUT2-like and SGLT1-like positive cells were also observed dispersed in the submucosa. Finally, a small population of SGLT1-like cells was detected in the brush border of the mucosa. Quantification of immunoreactive cells demonstrated that GLUT2-like cells are the most abundant (51.5%) of the three glucose transporters studied, followed by SGLT1-like cells (30.8%) and SGLT2-like cells (17.7%) (Fig. 1d). Apart from the mentioned bright and discrete cells, intestine immunostaining also revealed a broad faint signal in the apical membrane for SGLT1, and, to a lesser extent, for GLUT2 (Fig. 1a,b).

### Ghrelin, GOAT and GHS-R1a colocalize GLUT2, SGLT1 and/or SGLT2 in the goldfish intestine

Ghrelin-like (red; Fig. 2a–c, Supplementary Fig. S1), GOAT-like (red; Fig. 2g–i, Supplementary Fig. S2) and GHS-R1a (red; Fig. 2m–o, Supplementary Fig. S3) immunopositive cells were found scattered in the goldfish intestinal mucosa, adjacent to the lamina propria. A small proportion of submucosal cells also showed ghrelin-like (Fig. 2a, Supplementary Fig. S1), GOAT-like (Fig. 2g,h, Supplementary Fig. S2) and GHS-R1a-like (Fig. 2n, Supplementary Fig. S3) immunoreactivity. Ghrelin-like cells were almost equal in number to GLUT2-like cells (Fig. 2d), but considerably less abundant than SGLT1-like cells (Fig. 2e) and more widespread than SGLT2-like cells (Fig. 2f). The relative abundance of cells immunopositive for GOAT and SGLT2 was similar, but GOAT-like cells were greater in number in comparison to GLUT2-like and SGLT1-like cells (Fig. 2j–l). Finally, the relative number of GHS-R1a-like cells was higher, similar and lesser in comparison to GLUT2-like, SGLT1-like and SGLT2-like cells, respectively (Fig. 2p–r).

Fluorescence double staining showed the colocalization of ghrelin-like and GLUT2-like (yellow; Fig. 2a; Supplementary Fig. S1) and SGLT2-like (yellow; Fig. 2c; Supplementary Fig. S1) in a few mucosal cells. Specifically, 37.5% and 25% of the ghrelin-like cells coexpressed GLUT2 and SGLT2, respectively (Fig. 2d,f). No cells coexpressing ghrelin and SGLT1 were detected (Fig. 2b; Supplementary Fig. S1). GOAT-like immunoreactive cells were found to overlap with GLUT2 (yellow; Fig. 2g; Supplementary Fig. S2), but not SGLT1 (Fig. 2h; Supplementary Fig. S2) and SGLT2 (Fig. 2i; Supplementary Fig. S2), in some cells of the mucosa (28.6% of GOAT-like cells and 44.4% of GLUT2-like cells; Fig. 2j–l). Finally, GHS-R1a-like cells were found to colocalize with cells containing the three glucose transporters studied. These cells were also restricted to basal mucosal cells adjacent to the submucosa layer (yellow; Fig. 2m–o; Supplementary Fig. S3). Quantification demonstrated that 37.5% of the GLUT2-like, 25% of the SGLT1-like and 33.3% of the SGLT2-like immunoreactive cells coexpressed GHS-R1a (Fig. 2p–r). In all negative control immunostainings performed, no signal was detected in the slides treated only with secondary antibodies (Supplementary Fig. S4).

### Glucose upregulates the ghrelinergic system in goldfish intestine *in vivo*

**Figure 3:**
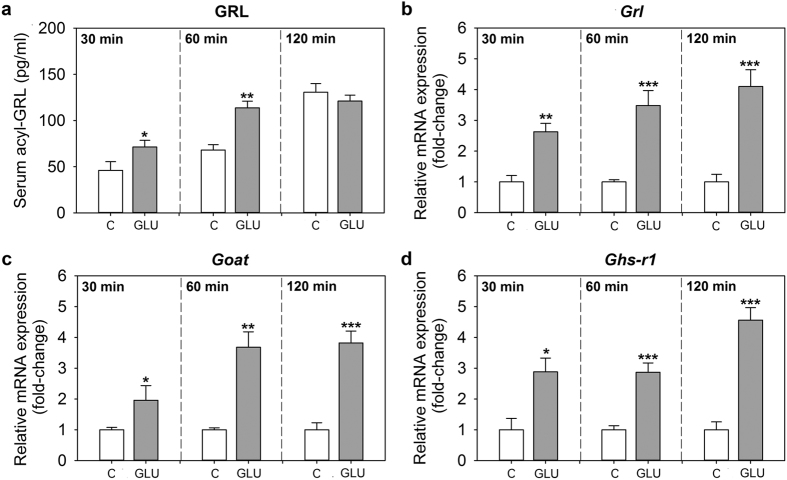
Effects of intraperitoneal administration of glucose (GLU) on circulating levels of acylated ghrelin (**a**) and on the expression of *preproghrelin* (**b**), *goat* (**c**) and *ghs-r1* (**d**) mRNAs in goldfish intestine. Fish were injected with saline alone or containing D-glucose (2 mg/g bw), and samples were collected at 30, 60 and 120 min post-injection. Circulating hormone levels and mRNA levels were quantified by ELISA and RT-qPCR, respectively. Data are presented as mean + SEM (n = 6 fish). Asterisks denote statistical differences between control and treated group assessed by t-test (*p < 0.05, **p < 0.01, ***p < 0.001). C, control.

Intraperitoneal (ip) administration of glucose in goldfish caused a significant increase in serum acylated ghrelin levels at 30 and 60 min, but not at 120 min, when compared to the saline-injected groups. The magnitude of the increases was of around 1.5- and 1.7-fold, respectively (Fig. 3a). The expression of *preproghrelin, goat* and *ghs-r1* mRNAs in the intestine was also upregulated by glucose at 30, 60 and 120 min post-injection, when compared to the control (Fig. 3b–d). In all cases, the highest increase in mRNA levels was observed at 120 min.

### Ghrelin increases GLUT2, SGLT1 and SGLT2 in goldfish intestine *in vivo* and *in vitro*

**Figure 4:**
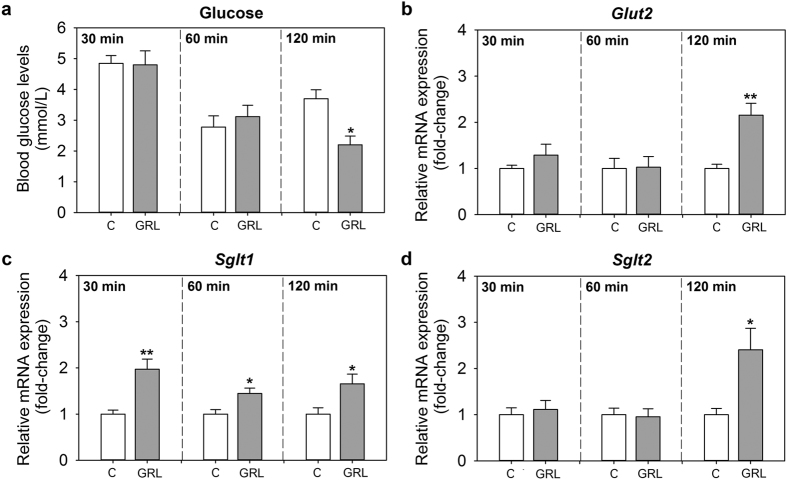
Effects of intraperitoneal administration of ghrelin (GRL) on blood glucose levels (**a**) and on the expression of *glut2* (**b**), *sglt1* (**c**) and *sglt2* (**d**) mRNAs in goldfish intestine. Fish were injected with saline alone or containing goldfish ghrelin (100 ng/g bw), and samples were collected at 30, 60 and 120 min post-injection. Gene expression levels were quantified by RT-qPCR. Data are presented as mean + SEM (n = 6 fish). Asterisks denote statistical differences between control and treated group assessed by t-test (*p < 0.05, **p < 0.01). C, control.

A single ip injection of ghrelin caused a significant decrease in blood glucose levels at 120 min post-injection when compared to the control group. No differences in glycaemia were observed between saline- and ghrelin-injected fish at 30 and 60 min post-injection (Fig. 4a). Intestinal expression of *glut2* and *sglt2* was 2-fold increased by ghrelin administration at 120 min, while remained unaltered at 30 and 60 min (Fig. 4b,d). Finally, ghrelin injection led to a 2-, 1.5- and 1.7-fold upregulation of *sglt1* mRNA levels in the intestine at 30, 60 and 120 min, respectively (Fig. 4c).

**Figure 5:**
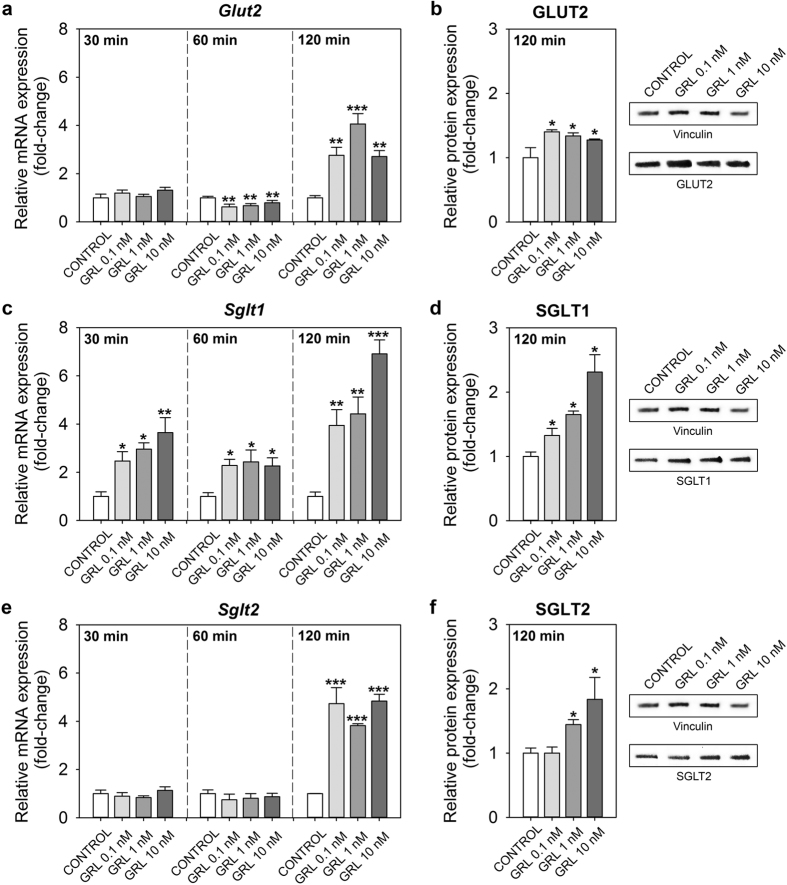
Effects of *in vitro* treatment with ghrelin (GRL) on the mRNA expression (left panel) and protein levels (right panel) of GLUT2, SGLT1 and SGLT2 in goldfish cultured intestine. (**a,c,e**) Concentration and time-dependent effects of ghrelin on glucose transporters gene expression. Cultured intestine was incubated with DMEM alone (control) or containing different concentrations of ghrelin (0.1, 1 and 10 nM) during 30, 60 and 120 min. Data obtained by RT-qPCR is shown as mean + SEM (n = 6 fish). Asterisks denote statistical differences between control and treated groups assessed by ANOVA and Student-Newman-Keuls post-hoc test (*p < 0.05, **p < 0.01, ***p < 0.001). (**b,d,f**) Protein levels of glucose transporters in cultured intestine treated with different concentrations of ghrelin (0.1, 1 and 10 nM) during 120 min. Data are presented as band density of GLUT2/vinculin, SGLT1/vinculin and SGLT2/vinculin (n = 3 fish). Representative blots from one fish are shown in image. Gel images shown here were cropped to show specific bands of expected size representing glucose transporters. Asterisks denote statistical differences between control and treated groups assessed by ANOVA and Student-Newman-Keuls post-hoc test (*p < 0.05).

*In vitro* exposure of intestinal portions to different concentrations of ghrelin resulted in a significant upregulation of *glut2* transcripts at 120 min, while a downregulation was observed at 60 min, with all ghrelin concentrations tested (0.1, 1 and 10 nM). No differences in the expression of this glucose transporter were observed after 30 min of treatment with ghrelin (Fig. 5a). Expression of *sglt1* mRNA was upregulated by all ghrelin concentrations tested at 30, 60 and 120 min post-incubation. The magnitude of the increases was of around 2.5-3-fold (at 30 min), 2.2-2.5-fold (at 60 min) and 4-7-fold (at 120 min) (Fig. 5c). Finally, ghrelin caused a 4-5-fold increase in *sglt2* mRNAs at 120 min, but no changes were observed at 30 and 60 min (Fig. 5e). Regarding protein levels, GLUT2 and SGLT1 were induced by *in vitro* treatment of intestinal portions with 0.1, 1 and 10 nM ghrelin (Fig. 5b,d). The higher concentrations of ghrelin (1 and 10 nM) also induced the expression of SGLT2 protein (Fig. 5f). Protein molecular weights of the transporters studied are: 57 kDa for GLUT2 and 73 kDa for SGLT1 and SGLT2.

### GHS-R1a and the PLC/PKC intracellular signal transduction pathways are involved in the ghrelin-induced upregulation of glucose transporter expression

**Figure 6:**
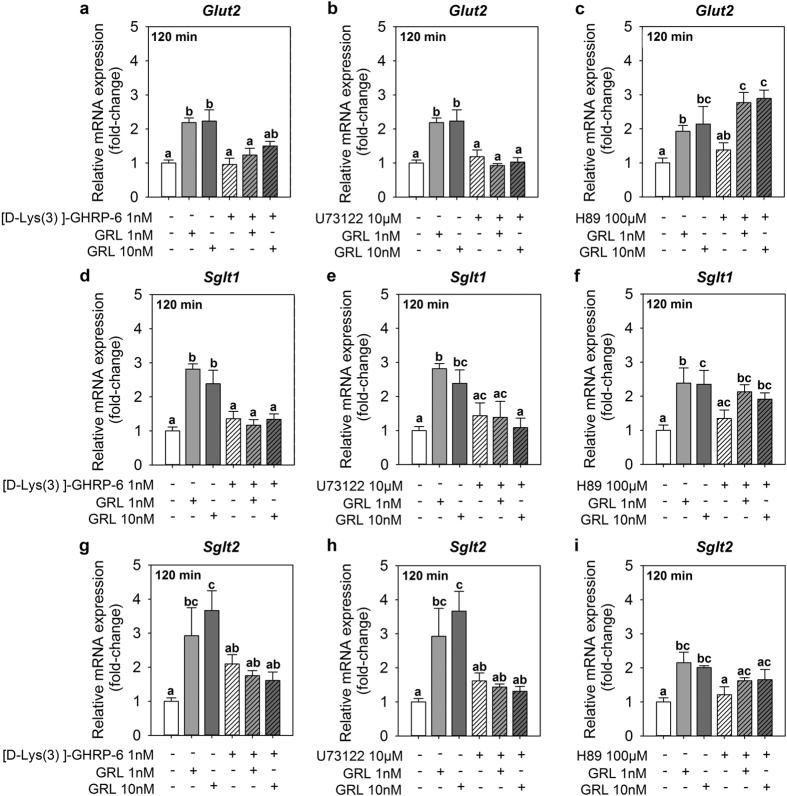
Relative expression of *glut2* (**a–c**), *sglt1* (**d–f**) and *sglt2* (**g–i**) mRNAs in goldfish cultured intestine treated with ghrelin (GRL) and either the GHS-R1a antagonist [D-Lys3]-GHRP-6 (left panel), the PLC inhibitor U73122 (middle panel) or the PKA inhibitor H89 (right panel) at 120 min post-incubation. The antagonist (1 nM) and inhibitors (10 μM for U73122 and 100 μM for H89) were added to the culture plates 15 min prior to the addition of ghrelin (1 and 10 nM). Data obtained by RT-qPCR are shown as mean + SEM (n = 6 fish). Different letters indicate statistical differences among groups assessed by ANOVA and Student-Newman-Keuls post-hoc test (p < 0.05).

The presence alone of the GHS-R1a antagonist [D-Lys3]-GHRP-6 in the intestinal *in vitro* preparations did not modify the expression of any of the glucose transporters tested at 120 min (Fig. 6a,d,g). The incubation of the preparations with the antagonist prior to the addition of ghrelin attenuated the increases in *glut2, sglt1* and *sglt2* mRNA expression induced by 1 and 10 nM ghrelin, resulting in expression values not statistically different from control groups (Fig. 6a,d,g).

The PLC inhibitor (U73122) and the PKA inhibitor (H89) by themselves did not modify the expression of glucose transporters in cultured intestine (Fig. 6b,c,e,f,h,i). Pre-incubation with U73122 completely abolished the induction of *glut2, sglt1* and *sglt2* expression by ghrelin at 120 min (Fig. 6b,e,h). Ghrelin was also unable to upregulate the expression of intestinal *sglt2* in the presence of H89 in the culture media (Fig. 6i). However, the ghrelin-induced upregulation of *glut2* and *sglt1* was not blocked by the pretreatment with H89 (Fig. 6c,f).

### Ghrelin stimulates the translocation of GLUT2, but not SGLT1 and SGLT2, into the plasma membrane of goldfish intestinal cells

**Figure 7:**
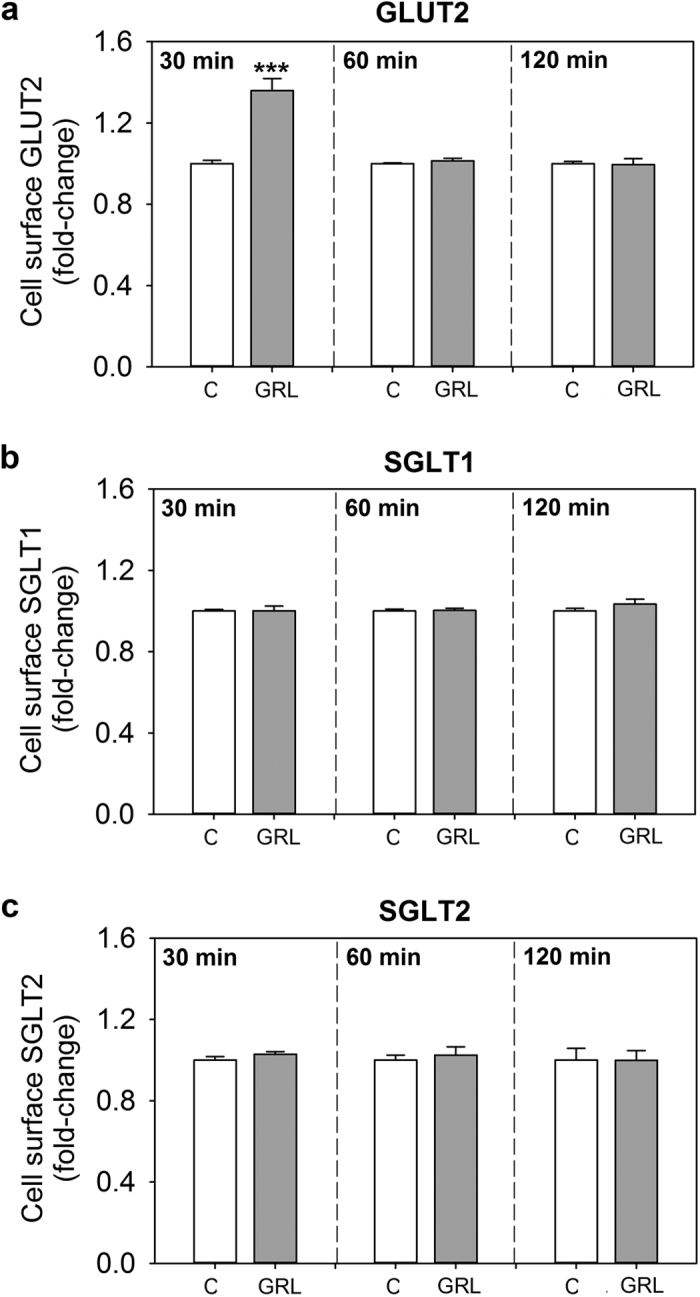
Levels of GLUT2 (**a**), SGLT1 (**b**) and SGLT2 (**c**) in the surface of goldfish intestinal cells treated with ghrelin (GRL) during 30, 60 and 120 min. The fraction of each glucose transporter at the cell surface was calculated as the ratio of surface to total cellular glucose transporter. Data are expressed as mean + SEM (n = 6 wells). Asterisks denote statistical differences between control and treated group assessed by t-test (***p < 0.001). C, control.

The amount of GLUT2 present at the surface of primary goldfish intestinal cells was 1.4-fold increased by 30 min of exposing the cells to culture media containing 10 nM ghrelin, when compared to cells treated with media alone. No effects were observed when the exposure time was 60 or 120 min (Fig. 7a). Ghrelin did not elicit any effect in the translocation rate of SGLT1 and SGLT2 at any of the time points tested (Fig. 7b,c).

## Discussion

This research characterized the cellular distribution of glucose transporters in goldfish intestine, and demonstrated for the first time its regulation by the gut-brain orexigenic hormone ghrelin. The presence of glucose transporters in the fish intestine has been previously studied using PCR techniques, having been demonstrated that the fish intestinal walls expresses the transporters GLUT2^13^,^47^ and SGLT1^15^. Furthermore, some studies using isolated enterocytes^48^ and intestinal membrane vesicles preparations^10^ have determined the transport rate and so the affinity of these two transporters in fish. The results of this study showing the presence of these two transporters in the goldfish intestine are in accordance with previous observations, and additionally report the presence of SGLT2 in the intestine. Besides, we offer additional information on their cellular distribution. Specifically, we observed the presence of GLUT2 in the basolateral border and of SGLT1 in the brush border of the mucosal cells, consistent with the putative location previously suggested for these two transporters within the fish enterocyte^48^, and in agreement with the classical model of intestinal glucose absorption in mammals: SGLT1 mediates glucose absorption from the intestinal lumen, whereas GLUT2 provides basolateral exit^3^,^49^. According to kinetics studies^10^,^48^, GLUT2 and SGLT1 in fish would function similar to mammals, transporting glucose with low and high affinity, respectively. However, not only GLUT2 but also SGLT1 and SGLT2 were detected in the basolateral border of the goldfish mucosal cells, suggesting that the three transporters might participate in the transport of glucose from cytosol to the blood in goldfish.

Double immunostaining of goldfish intestinal sections showed that GLUT2 and SGLT2 are present in mucosal cells expressing ghrelin. Some GLUT2-positive cells were also observed to contain GOAT, the enzyme responsible for its acylation. Although glucose transporters are mainly present in enterocytes, the presence of some of them, especially SGLT1, has been reported in enteroendocrine cells of both mammals^50^,^51^ and fish^14^,^15^. It has been suggested that this transporter, together with taste receptors and other G protein-coupled receptor, are present in some enteroendocrine cells as part of the machinery that interacts with chemical food components to trigger the release of gut peptides, such as cholecystokinin, peptide YY, GIP and GLP-1^50^,^51^. Furthermore, Gorboulev and coworkers^52^ reported that the secretion of GIP and GLP-1 is stimulated by fat ingestion in both wild‐type and SGLT1‐deficient mice, but not by glucose in SGLT1‐deficient mice, clearly showing that SGLT1 plays a critical role in incretins secretion in response to glucose *in vivo*. Results from the present study show that glucose plays an important stimulatory role in the secretion of ghrelin, as well as in the intestinal mRNA expression of *preproghrelin, goat* and *ghs-r1*, in accordance with previous observation in tilapia^53^. The fact that the glucose modulation of the ghrelinergic system was observed at all the time points tested indicates a high sensitivity of the ghrelinergic system to glucose and points to a rapid and maintained action of this monosaccharide on the system. The observed stimulatory effect of glucose on ghrelin might be governed by first an intestinal sensing of the monosaccharide, mechanism by which glucose transporters are known to play a role^54^. In the present study, the fact that GLUT2 and SGLT2, but not SGLT1, are coexpressed with ghrelin in the same cells suggests a role for GLUT2 and/or SGLT2, but not SGLT1, in ghrelin release from fish goldfish gut endocrine cells.

Using double immunofluorescence, we also observed that a considerable percentage (37.5%, 25% and 33.3%, respectively) of GLUT2-, SGLT1- and SGLT2-positive intestinal cells coexpress the ghrelin receptor GHS-R1a, suggesting that they are susceptible to modulation by ghrelin. To the best of our knowledge, no studies have previously reported a role for ghrelin in the modulation of glucose transporters in the intestine of any vertebrate. A role for ghrelin in this function has been shown, however, in other tissues, such as the hypothalamic astrocytes^30^, the white adipocytes^31^, and the cardiomyocytes^32^ of mammals, and the hypothalamus and hindbrain of fish^34^. Using both *in vivo* and *in vitro* approaches, here we demonstrated an important stimulatory role for ghrelin in the gene expression of the three transporters in the intestine of goldfish. Protein levels of the transporters were also upregulated by ghrelin *in vitro*. While protein was not quantified in the *in vivo* study, it is highly likely that the increases in mRNA expression observed *in vivo* (as well as in the rest of the experiments) correspond with an increase in protein. Almost all the changes at the mRNA level observed *in vitro* extended to proteins. The fact that ghrelin modulates glucose transporters suggests an important role for this hormone on intestinal glucose transport. A hormonal regulation of this process in fish has been suggested previously in a study showing that glucagon, GLP-1, glucocorticoids (dexamethasone) and catecholamines (isoproterenol) significantly increase the rate of brush-border glucose transport in the enterocytes of the black bullhead^48^. Further studies would be needed to investigate the physiological significance of both anabolic (ghrelin) and catabolic (the others) hormones stimulating glucose absorption. Among the three transporters tested in the present study, SGLT1 seems to be the more sensitive to ghrelin, as its expression was upregulated during all the time points studied. Expression of GLUT2 and SGLT2, however, was only affected at 120 min post-injection/post-incubation. This indicates a chronology in the actions of ghrelin on the different glucose transporters, which might be in accordance with first facilitating the absorption of glucose from the intestinal lumen (which is mainly mediated by SGLT1) and then assisting in its transport to the blood.

While ghrelin seems to enhance intestinal glucose absorption, *in vivo* ghrelin treatment resulted in a decrease in blood glucose levels. This is likely due to similar stimulatory effects of ghrelin on glucose transporters in other locations in order to facilitate glucose absorption into them, so determining an eventual decrease in circulating glucose. One such location seems to be the liver, as supported by the fact that the expression of *glut2* and *sglt1* are also upregulated by ghrelin in this tissue (Supplementary Fig. S5). The fact that the stimulatory effects of ghrelin on glucose transporters in both the intestine and liver are observed within the same time window suggests that this hormone is simultaneously promoting glucose absorption from the intestinal lumen to the circulation and glucose uptake from the blood to storage locations, thereby preventing a condition of hyperglycemia. The effects of ghrelin on glycemia seem, however, controversial within the literature, as previous studies have shown that administration of this hormone does not alter circulating glucose levels in rainbow trout^34^ while produces a significant increase in tilapia^55^. These species-specific discrepancies might be dependent on different dietary habits, although more studies would be needed to elucidate the species- and tissue-specific effects of ghrelin on metabolic partitioning, and glucose production in fishes.

Results presented here demonstrate that all of the observed effects of ghrelin on gene expression are mediated by its receptor GHS-R1a, as all the inductions in expression were abolished by the use of the receptor antagonist [D-Lys3]-GHRP-6. Which intracellular signaling pathway(s) does this receptor trigger to exert ghrelin actions on glucose transporters? Literature shows that the GHS-R1a is classically linked to a signal transduction mechanism involving an increase in the cytosolic free Ca^2+^ concentration ([Ca^2+^]_i_), especially via the PLC/PKC and the AC/PKA pathways^39^,^40^,^41^,^56^. These two signal transduction mechanisms have been reported to mediate various actions of ghrelin/GHS-R1a. For instance, in mammals, the PLC/PKC pathway has been involved in the release of growth hormone (GH) from pituitary^57^, whereas the AC/PKA pathway has been reported to mediate the interaction between ghrelin and neuropeptide Y neurons in the arcuate nucleus^56^. In fish, both the PLC/PKC and the AC/PKA pathways were involved in the ghrelin-induced GH and gonadotrophin release from goldfish pituitary cells^41^ and the actions of ghrelin on clock gene expression in goldfish (Sánchez-Bretaño *et al*., *under review*). In this study, all ghrelin-evoked gene expression inductions are blocked by the use of a PLC inhibitor (U73122), but not by the PKA inhibitor (H89). These results provide reliable evidence indicating that the PLC/PKC pathway, but likely not the AC/PKA pathway, is the responsible for the intracellular signal transduction that will eventually modulate the expression of glucose transporters after ghrelin binds to GHS-R1a. While we are providing evidence that the AC/PKA pathway does not mediate such ghrelin effects, present results do not exclude the possible involvement of other signaling pathways. Indeed, GHS-R1a has been reported to trigger the AMPK, MAPK or mTOR intracellular cascades to mediate some ghrelin actions^39^.

To deepen the knowledge of the mechanisms underlying ghrelin actions on intestinal glucose transporters, our last objective was to determine whether ghrelin modulates transporter translocation into the cell surface. Immunoblotting analysis in goldfish primary intestinal cells revealed that ghrelin increases the amount of GLUT2, but not SGLT1 and SGLT2, at the plasma membrane. This effect was only observed at 30 min post-incubation with ghrelin, but not after longer exposure times, indicating that this modulatory action of ghrelin on glucose transporter translocation is time-specific. The translocation of GLUT2 from intracellular vesicles into the plasma membrane has been previously reported in *in vivo* perfusion studies in rats as a mechanism to allow bulk absorption of glucose by facilitated diffusion after high luminal glucose loads^3^,^5^. This rapid insertion of GLUT2 into the plasma membrane has been shown to be modulated by some hormones, such as glucagon-like peptide 2 (GLP-2), which was reported to promote the translocation of this transporter into the rat jejunal brush-border membrane^58^. Findings from the present study represent the first demonstration that ghrelin is able to cause the translocation of GLUT2 in intestinal cells, likely resulting in an improved ability of these cells to take up glucose.

**Figure 8:**
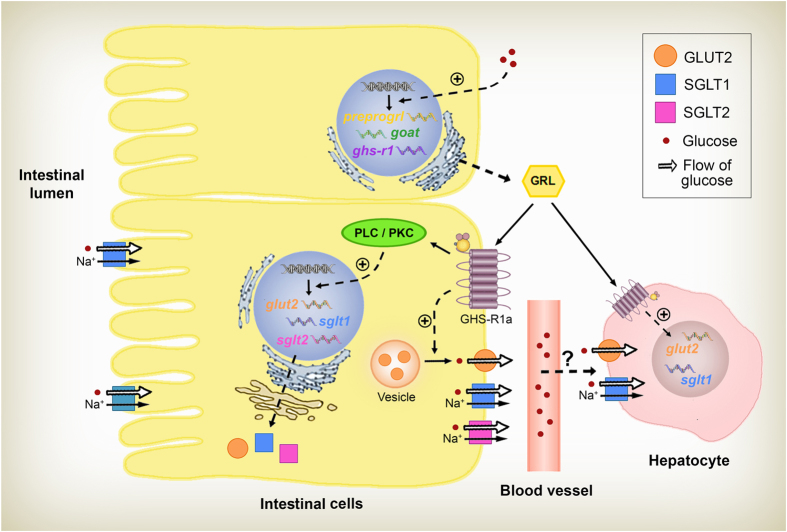
Schematic representation of the proposed ghrelinergic regulation of glucose transport machinery in the intestinal cells of goldfish. Mucosal cells of the goldfish intestine contain the glucose transporters SGLT1 in the apical or brush border, and GLUT2, SGLT1 and SGLT2 in the basolateral border. Ghrelin, via the GHS-R1a and the PLC/PKC pathway, would modulate the gene transcription in the nucleus, enhancing the expression of *glut2, sglt1* and *sglt2*. This increase in the number of transcripts would also be reflected in the amount of each of the proteins. Additionally, ghrelin would stimulate the translocation of GLUT2 from intracellular vesicles to the surface of intestinal cells, all of this to facilitate the absorption of glucose. All this ghrelinergic action might be initiated by a glucose-induced upregulation of the intestinal ghrelinergic system. We furthermore hypothesize that ghrelin would facilitate glucose uptake into hepatocytes *via* GLUT2 and/or SGLT1 transporters. GHS-R1a, growth hormone secretagogue receptor 1a; GRL, ghrelin; PKC, phosphokinase C; PLC, phospholipase C.

In summary, our data indicate that an important crosstalk between ghrelin and cellular glucose transport occurs in the intestine of goldfish. A proposed model for this crosstalk is summarized in Fig. 8. First, an increased expression of all components of the ghrelinergic system in the intestine and ghrelin secretion after glucose treatment indicates that this monosaccharide stimulates ghrelin. It is plausible that ghrelin would respond to an increase in glucose by upregulating the expression of its transporters GLUT2, SGLT1 and SGLT2, as well as by promoting the translocation of GLUT2 into the plasma membrane of intestinal cells, all these for facilitating the absorption of glucose. The involvement of two parameters related to glucosensing in fish (GLUT2 and SGLT1; 54) in this sequence of actions could point to a glucosensing response of intestinal cells modulated by ghrelin: intestinal cells would detect changes in glycaemia and respond to them by producing higher levels of ghrelin, which would in turn modulate glucosensing via an action on glucose transporters. Apart from affecting intestinal glucose transport, ghrelin would also facilitate the uptake of glucose from the blood to the hepatic cells, likely helping to avoid a situation of hyperglycemia and to store glucose. This ghrelinergic regulation of glucose transport would operate during feeding in order to improve nutrient utilization, thus being initiated by the high levels of ghrelin known to be in circulation before a meal^59^. Additionally, it has been reported that the ghrelin-induced activation of the glucosensor system in the brain of rainbow trout would eventually lead to an effect on food intake^34^. It is thereby possible that this ghrelinergic regulation of intestinal glucose transport relate to the effects of ghrelin on food intake in fish. On a larger scale, we can also hypothesise that this regulatory mechanism would help fish to cope with the varied food availability situations they have to face in their natural environments. Overall, these novel results demonstrate that ghrelin plays an important role in glucose transport across the goldfish intestinal walls, thus participating in glucose homeostasis in fish. Further investigations would be needed to test the effects of ghrelin on the transporter activity and to determine whether this peptide is also a modulator of glucose transport in other key organs.

## Methods

### Animals

Goldfish (*Carassius auratus*), with a body weight (bw) of 5 ± 1 g (for immunohistochemistry) or 32 ± 8 g (for *in vivo* and *in vitro* studies), were obtained from a commercial supplier (Aquatic Imports, Calgary, AB, Canada). Fish were housed in either 300 L aquaria with filtered fresh water at 20 ± 2 °C and continuous aeration (for immunohistochemistry and *in vitro* studies), or 10 L aquaria (n = 3/aquarium) with a constant flow of temperature-controlled water (21 ± 2 °C) (for *in vivo* studies). Fish were maintained under a 12 h light:12 h darkness (12 L:12D) photoperiod (lights on at 07:00 h), and fed daily at 10:00 h with food from a commercial pellet diet for goldfish (1% bw; Goldfish granules, Aqueon, Franklin, WI, USA). All fish studies adhered to the Canadian Council of Animal Care guidelines, and research protocols were approved by the Animal Research Ethics Board of the University of Saskatchewan (Protocol Number 2012-0082).

### Reagents

D-glucose was obtained from Fisher Scientific (Ottawa, ON, Canada) and prepared in saline (Catalog # JB1323, Baxter, Mississauga, Canada) at a concentration of 2 mg/10 μl saline. The acylated 19 amino acid isoform of goldfish ghrelin (GTS(Octanoyl)FLSPAQKPQGRRPPRM) was custom synthesized by GenScript (Piscataway, NJ, USA). For *in vivo* studies, peptide was reconstituted in distilled water and then prepared in saline at a concentration of 100 ng/10 μl saline. For *in vitro* studies, ghrelin was reconstituted in distilled water at a stock concentration of 100 μM. The GHS-R1a ghrelin receptor antagonist [D-Lys3]-GHRP-6 and the PLC inhibitor U73122 (both from Sigma-Aldrich, Oakville, ON, Canada) stock solutions were dissolved in absolute ethanol at a concentration of 10 mM. Stock solution of the PKA inhibitor H89 (Sigma-Aldrich) was prepared in distilled water at a concentration of 10 mM. All the stock solutions (ghrelin, [D-Lys3]-GHRP-6, U73122 and H89) were diluted in Dulbecco’s Modified Eagle Medium (DMEM) supplemented with 44 mM sodium bicarbonate, 1% penicillin-streptomycin and 0.05% gentamicin (DMEM+) to reach the required experimental concentrations just before use. The DMEM+ used for control and ghrelin-treated groups in the experiments using [D-Lys3]-GHRP-6 and U73122 (reconstituted in a ethanol) was supplemented with the same amount of ethanol (0.05%) to minimize possible deviations.

### Immunohistochemistry

Samples from anterior intestine (1–2 cm after the intestinal bulb) were collected as previously described^60^ and processed (dehydrated and embedded in paraffin) at the Prairie Diagnostic Services, University of Saskatchewan. Paraffin blocks were then sectioned at 7 μm thickness, and transversal sections were mounted onto Superfrost slides (Thermo Fisher Scientific, Waltham, MA, USA). The protocol for IHC was performed as previously described^61^ with slight modifications. Briefly, after deparaffination, rehydration, washing and blocking, sections were incubated overnight with the corresponding primary antibody/es (1:200 dilution) at room temperature. In general, a mixture of two antibodies was prepared for colocalization studies, except for the antibodies used for GOAT, GHS-R1a, SGLT1 and SGLT2 which are all raised in the same host species. In these cases, colocalization was approached by staining consecutive sections with the different antibodies separately. Primary antibodies used were: mouse monoclonal to ghrelin, Catalog # ab57222; rabbit polyclonal to GOAT, Catalog # ab140889; rabbit polyclonal to GHS-R1a, Catalog # ab95250; goat polyclonal to GLUT2, Catalog # ab111117; rabbit polyclonal to SGLT1; Catalog # ab14686; rabbit polyclonal to SGLT2; Catalog # ab85626 (all from Abcam, Toronto, ON, Canada). Since heterologous antibodies were used here, it is likely that a certain degree of non-specificity exists in our findings. Therefore, the suffix “-like” was used to refer to immunostaining obtained. The following day, sections were washed and subsequently incubated for 1 h at room temperature with a mixture of one (single stainings) or two (double staining) secondary antibodies (1:2000 dilution each). Secondary antibodies used were: donkey anti-goat IgG Alexa Fluor 488, goat anti-rabbit IgG Alexa Fluor 488, goat anti-mouse IgG Alexa Fluor 594 (all from Invitrogen, Burlington, Canada), goat anti-mouse Alexa Fluor 647, donkey anti-rabbit Alexa Fluor 647 (both from Abcam), and Texas Red anti-rabbit IgG (Vector Laboratories, Burlington, Canada). A separate set of negative control slides were only treated with the secondary antibodies. All primary and secondary antibodies were diluted in antibody diluent reagent (Dako, Mississauga, ON, Canada). Finally, slides were mounted using VECTASHIELD Mounting Medium containing 4′,6-diamidino-2-phenylindole (DAPI; Vector Laboratories, Burlington, ON, Canada) and assessed using either a Nikon Eclipse Ti-Inverted fluorescence microscope (Nikon Instruments, Melville, USA) or a Leica TCS SP5 confocal microscope (Leica Biosystems, Concord, ON, Canada). Micrographs were adjusted linearly for light and contrast using Photoshop CS6 (Adobe Systems Inc., San Jose, USA).

For the quantification of immunopositive cells, the number of cells in the mucosa and submucosa immunoreactive for ghrelin, GOAT or GHS-R1a (red), GLUT2, SGLT1 or SGLT2 (green), or colocalizing both ghrelin/GOAT/GHS-R1a and GLUT2/SGLT1/SGLT2 (yellow) were counted in all sections assessed (n = 3 sections). The abundance of the different glucose transporters in the goldfish intestine was determined by calculating the percentage of the number of positive cells for each of the transporters in relation to the total number of immunoreactive cells to any of the transporters (GLUT2 + SGLT1 + SGLT2). In the colocalization studies, the total number of cells counted for ghrelin, GOAT or GHS-R1a alone and for GLUT2, SGLT1 or SGLT2 alone was plotted in pie-charts, showing the relative abundance of ghrelin/GOAT/GHS-R1a and GLUT2/SGLT1/SGLT2 in each of the assessed combinations. Then, to calculate the percentage of ghrelin/GOAT/GHS-R1a cells that colocalizes GLUT2/SGLT1/SGLT2 and *vice versa*, the number of cells positive for the two peptides was calculated as a percentage of the total number of ghrelin/GOAT/GHS-R1a cells (for the first case) or of the total number of GLUT2/SGLT1/SGLT2 (for the second case). These percentages were indicated and shadowed in the corresponding pie-charts.

### *In vivo* studies

#### Effects of glucose administration on the ghrelinergic system

Goldfish (n = 6 fish/group) were anesthetized in 0.5% tricaine methanesulfonate (TMS; Syndel Laboratories, Vancouver, BC, Canada) and ip injected with saline alone (control) or containing D-glucose (2 mg/g bw). Glucose dose was chosen based on previous studies^62^. Injections were carried out at 10:00, in 24-h fasted fish, and were performed close to the ventral midline posterior to the pelvic fins. At 30, 60 and 120 min post-injection, fish were anesthetized and samples of blood were collected from the caudal vein for quantifying ghrelin levels in serum. Fish were sacrificed by spinal dissection and samples of anterior intestine (approximately 1 cm after the intestinal bulb) were collected, quickly frozen in liquid nitrogen and stored at −80 °C until quantification of mRNA levels (see *Real-time quantitative PCR* section).

#### Effects of ghrelin administration on intestinal glucose transporters

Goldfish (n = 6 fish/group) were anesthetized in 0.5% TMS and ip injected with saline alone (control) or containing goldfish ghrelin (100 ng/g bw, 3 μM). Injections were performed as described for the previous experiment. At 30, 60 and 120 min, fish were anesthetized and a spinal transection was carried out, followed by monitoring of blood glucose levels using a glucometer (One-Touch Ultra2; Johnson and Johnson Co., Burnaby, BC, Canada)^63^. Samples of anterior intestine were collected for gene expression analysis (see *Real-time quantitative PCR* section).

### *In vitro* organotypic studies

#### Concentration- and time-response effects of ghrelin on glucose transporter expression

Tissue culture was performed as previously described for goldfish^64^ with slight modifications. Briefly, intestinal portions (1–2 mm width; approximately 20 mg/portion) from the anterior intestine (from the end of the intestinal bulb until the J-loop) of 24 h-fasted goldfish (n = 6) were immersed in DMEM supplemented 10% penicillin-streptomycin and 0.5% gentamicin for 1 min, and then distributed in different wells of sterile culture 24-well multidish plates. Plates were preincubated for 2 h in 1 mL of DMEM+ at 23 °C under an atmosphere of 5% CO_2_ and 95% O_2_ for stabilization. Then, medium was replaced by 1 mL of fresh DMEM+ alone (6 wells, each well loaded with tissues from a different fish) or DMEM+ containing ghrelin (0.1, 1 or 10 nM; 6 wells each), and plates were incubated for 30, 60 or 120 min. At the end of each culture time, intestine samples were collected and stored at −80 °C until total RNA or protein was extracted (See *Real-time quantitative PCR* and *Western Blot* sections).

#### Involvement of the GHS-R1a ghrelin receptor and the PLC-PKC and AC-PKA pathways on ghrelin action

Intestine portions from 6 fish were prepared in 24-well multidish plates as described above. After the 2 h stabilization period, medium was replaced by 500 μl of fresh DMEM+ containing either the respective vehicle, the ghrelin receptor antagonist ([D-Lys3]-GHRP-6; 2 nM) or one of the inhibitors (U73122; 20 μM, or H89; 200 μM). After 15 min, 500 μl of DMEM+ containing the respective vehicle or ghrelin (2 or 20 nM) were added to the corresponding wells. Final concentrations of drugs in each well were: 1 nM for [D-Lys3]-GHRP-6, 10 μM for U73122, 100 μM for H89 and 1 nM and 10 nM for ghrelin. Plates were incubated for 120 min, before samples were collected for gene expression determination.

### *In vitro* studies using primary intestinal cells

#### Isolation of intestinal cells from goldfish

Protocol for isolating intestinal cells was adapted from the protocol described by El-Sabry and colleagues^65^ in rainbow trout. The intestine from goldfish (n = 5) was removed, freed from mesenteric fat, and rinsed three times in autoclaved deionised water. Tissue was incubated twice in DMEM containing 44 mM sodium bicarbonate, 2% penicillin-streptomycin and 0.1% gentamicin for 1 h at 4 °C. The intestine was then longitudinally opened, cut into 1 cm^2^ pieces and incubated in collagenase (Sigma-Aldrich) in DMEM+ (1 mg/mL) for 24 h at 4 °C. The following day, after the intestine pieces were shaken and the enterocytes were detached from the mucosa using a Pasteur pipette, the resulting intestinal cell suspension was filtered through a 30 μm mesh, collected in tubes and centrifuged (5 min at 3000 g). The pellet containing the intestinal cells was washed three times in DMEM+ and then resuspended in DMEM. Intestinal cells were plated onto gelatin coated 24-well plates at a concentration of 1 × 10^6^ cells/mL. Cells were grown in DMEM+ supplemented with 5% fetal bovine serum (FBS) at 23 °C under an atmosphere of 5% CO_2_ and 95% O_2_. The culture medium was changed every day to remove non-adherent cells. Viable intestinal cells were round and characterized by smooth non-folded cell membrane, and they typically achieved confluency on the third day.

#### Glucose transporter translocation assay

Translocation assay was performed in confluent cells as described by Díaz and colleagues^66^. Intestinal cells were serum deprived for 3–5 h and subsequently incubated with DMEM+ alone (control) or containing goldfish ghrelin (10 nM) for 30, 60 and 120 min. Medium was then removed by washing with ice-cold PBS supplemented with 1 mM CaCl_2_ and 1 mM MgCl_2_. Briefly, to label the amount of GLUT2, SGLT1 and SGLT2 in the cell surface, cells were blocked, and incubated with primary antibody against each of the glucose transporter (1:500 dilution; see *Immunohistochemistry* section for information on antibodies used). Cells were then washed and the fixative was immediately neutralized with glycine. To label the total cellular amount of each of the glucose transporters, a separate set of intestinal cells were first fixed, quenched in glycine, and then permeabilized. After blocking, cells were incubated with GLUT2/SGLT1/SGLT2 antibody solution (1:500 dilution), and washed. Both cell surface and total cellular GLUT2/SGLT1/SGLT2-bound antibodies were probed by incubation with HRP-conjugated secondary antibodies (Bio-Rad) for 1 h at 4 °C, followed by detection of bound secondary antibodies using *o*-phenylenediamide (OPD; Sigma-Aldrich) and absorbance reading at 450 nm. Translocation was quantified as described by Wang and coworkers^67^ using standard curves generated with each of the HRP-conjugated secondary antibodies. The fraction of GLUT2/SGLT1/SGLT2 at the cell surface was expressed as the ratio of surface to total cellular glucose transporter.

### Quantification of serum acylated ghrelin

Serum levels of acylated ghrelin were measured using a fish ELISA kit (Catalog # MBS034979, Mybiosource, San Diego, CA, USA) according to the manufacturer. The sensitivity of assay was 5.0 pg/mL, and the detection range was 31.2–1000 pg/mL. The amount of target peptide was determined by using a cubic regression curve-fit.

### Real-time quantitative PCR

Isolation of total RNA, synthesis of cDNA and real-time quantitative PCRs were performed as described in Ramesh *et al*.^68^. The specific primer sequences used for target genes, and reference gene (*β-actin*) are shown in Table 1 and were ordered from IDT (Toronto, ON, Canada). Primers used for quantifying ghrelin receptor were designed in a region conserved between the *ghs-r1a1* and *ghs-r1a2* sequences^69^, so PCR products correspond to the sum of all *ghs-r1* mRNA isoforms. RT-qPCR cycling conditions consisted of an initial step of 95 °C for 3 min, and 35 cycles of 95 °C for 10 sec and 60 °C (for *β-actin, goat* and *ghs-r1*), 58 °C (for *glut2, sglt1* and *sglt2*) or 56 °C (for *preproghrelin*) for 30 sec. All runs were performed using a CFX Connect Real-Time System (Bio-Rad). The 2-ΔΔCt method^70^ was used to determine the relative mRNA expression.

### Western Blot

For Western blot analysis we chose the concentrations and time in which ghrelin exerts the most significant inductions in mRNA expression. Protein extraction and quantification, and Western blot protocol were performed as previously described^68^. Briefly, the samples (containing 30 μg protein) were boiled, electrophoresed and transferred to a nitrocellulose membrane (Bio-Rad). After blocking, target proteins within the membrane were detected by overnight incubation in 1x RapidBlock™ solution containing specific primary antibody (see *Immunohistochemistry* section for information on antibodies used). Vinculin protein was used for normalization and was detected using rabbit antiserum directed against mouse vinculin (1:2000 dilution; Catalog ≠ ab129002, Abcam). As secondary antibodies, goat anti-rabbit or rabbit anti-goat IgG (H + L) HRP conjugate (Bio-Rad) diluted 1:3000 were used. Protein were visualized using Clarity™ Western ECL substrate (Bio-Rad) in a ChemiDoc™ MP imaging system (Bio-Rad) with chemiluminescence detection. Blot images were plotted using ImageJ software and band density of vinculin was used to normalize glucose transporters protein density.

### Statistics

Statistical differences between groups were assessed using either t-test (for comparisons between two groups) or one-way ANOVA followed by Student-Newman-Keuls multiple comparison test (for comparisons among multiple groups), after data were checked for normality and homogeneity of variance. Data that failed one of these requirements were log-transformed and re-checked. Significance was assigned when p < 0.05. All analyses were carried out using SigmaPlot version 12.0 (Systat Software Inc., San Jose, CA, USA) statistics package.

## Additional Information

**How to cite this article**: Blanco, A. M. *et al*. Ghrelin Facilitates GLUT2-, SGLT1- and SGLT2-mediated Intestinal Glucose Transport in Goldfish (*Carassius auratus*). *Sci. Rep.* **7**, 45024; doi: 10.1038/srep45024 (2017).

**Publisher's note:** Springer Nature remains neutral with regard to jurisdictional claims in published maps and institutional affiliations.

## Supplementary Material

Supplementary Information

## Figures and Tables

**Table 1:** Primers used in this study for quantifying gene expression by RT-qPCR.

Gene	GenBank accession number	Primer sequence (5′ to 3′)	Product size (bp)
*preproghrelin*	AF454389.1	F: ATTCAGAGTGTTGTCGTAR: AGGAAAGAGCACATAAGA	103
*goat*	KT726983	F: ATTGCTGTTCTTCAGTGCCGR: TGTACAAGTGCCAGACGGTT	119
*ghs-r1*	AB504275.1/AB504276.1	F: ATTCGAGCACCCGGTCAACAR: TCCAGGGGCATGCAGAGAAA	207
*glut2*	DQ098687.1	F: TGTGCTGTGGCCATGACR: CCAGGTCCGATCTCAAAGAA	113
*sglt1*	JN867793.1	F: GATCGTGACCATGCCAGAGR: TTTAGTCCCAGAGCCTGGTT	156
*sglt2*	BC067629.1	F: GCACCTTGTTCACCATGGACATR: ACCACTCTGGGCTGCCTG	146
*β-actin*	AB039726.2	F: CAGGGAGTGATGGTTGGCAR: AACACGCAGCTCGTTGTAGA	168

F, Forward primer; R, Reverse primer.
