# Supplementary material for: Ghrelin Facilitates GLUT2-, SGLT1- and SGLT2-mediated Intestinal Glucose Transport in Goldfish (Carassius auratus)
Source: Sci Rep. 2017 Mar 24;7:45024. doi: 10.1038/srep45024 (PMC5364492; doi:10.1038/srep45024)
Supplement: Supplementary Information [file srep45024-s1.pdf]

Ghrelin Facilitates GLUT2-, SGLT1- and SGLT2-mediated Intestinal Glucose Transport in Goldfish (*Carassius auratus*)

Ayelén Melisa Blanco[1,2], Juan Ignacio Bertucci[2,3], Naresh Ramesh[2], María Jesús Delgado[1], Ana Isabel Valenciano[1], Suraj Unniappan[2*]

[1] Departamento de Fisiología (Fisiología Animal II), Facultad de Biología, Universidad Complutense de Madrid, Madrid, Spain.

[2] Laboratory of Integrative Neuroendocrinology, Department of Veterinary Biomedical Sciences, Western College of Veterinary Medicine, University of Saskatchewan, Saskatoon, Saskatchewan, Canada.

[3] Instituto de Investigaciones Biotecnológicas-Instituto Tecnológico Chascomús, Buenos Aires, Argentina.

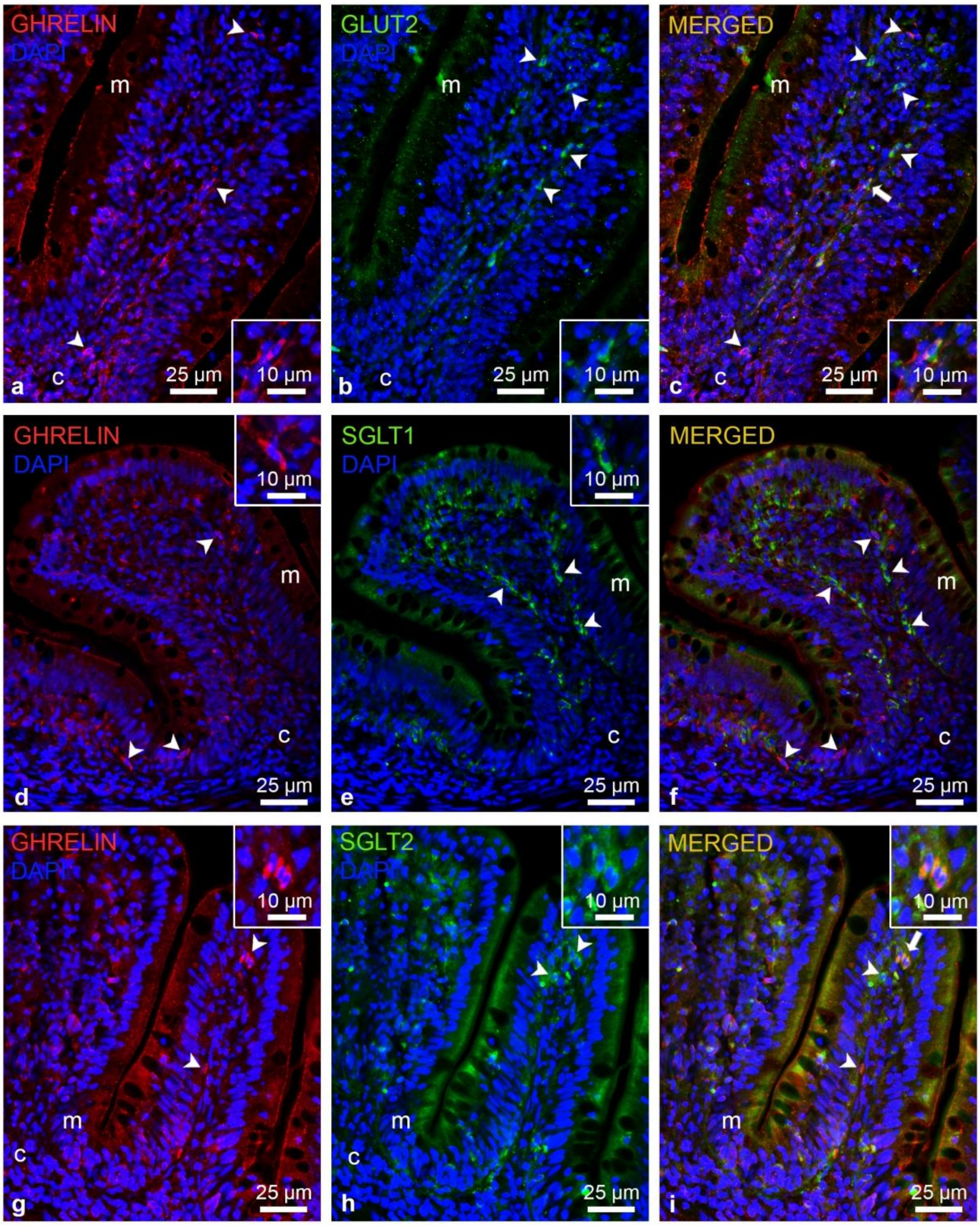

Supplementary Figure S1. Ghrelin-like and GLUT2-like, SGLT1-like and SGLT2-like immunoreactivity in goldfish intestine detected by immunohistochemistry and assessed by fluorescence miscroscope. Transversal representative sections of intestine showing ghrelin-like (a, d, g; red) and GLUT2-like (b; green), SGLT1-like (e; green) or SGLT2-like (h; green) immunoreactivity, and merged images of ghrelin and each of the glucose transporters (c, f, i; yellow). All images are merged with DAPI showing nuclei in blue. Arrowheads indicate cells stained with either ghrelin or GLUT2, SGLT1 or SGLT2, and solid arrows show cells that colocalize both ghrelin and each of the glucose transporters. A magnified image of representative cells positive to ghrelin and/or the glucose transporters is shown in square inset in each figure. Scale bars (µm) are indicated in each image. c, connective tissue (lamina propia + submucosa); m, mucosa.

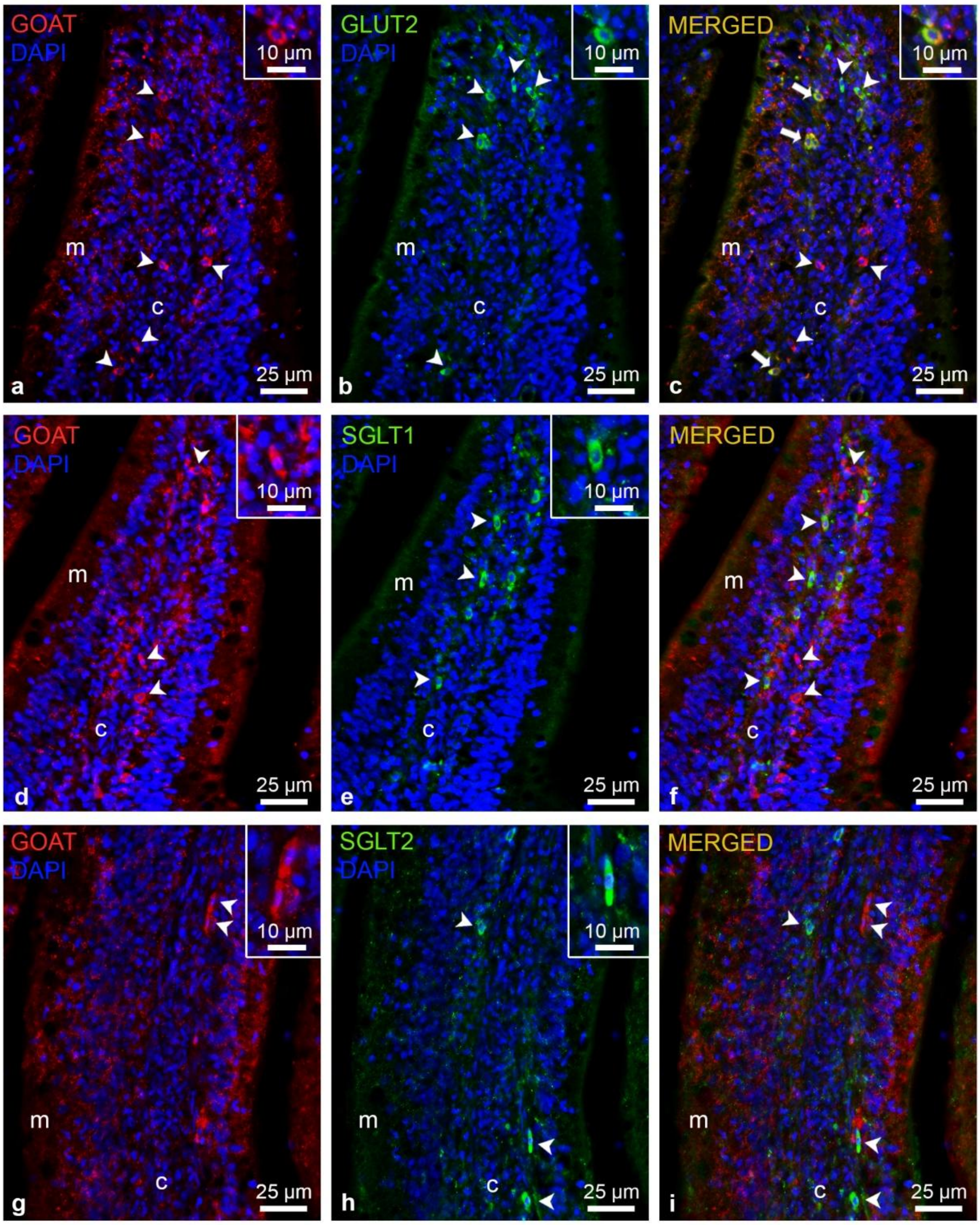

Supplementary Figure S2. GOAT-like and GLUT2-like, SGLT1-like and SGLT2-like immunoreactivity in goldfish intestine detected by immunohistochemistry and assessed by fluorescence miscroscope. Transversal representative sections of intestine showing GOAT-like (a, d, g; red) and GLUT2-like (b; green), SGLT1-like (e; green) or SGLT2-like (h; green) immunoreactivity, and merged images of GOAT and each of the glucose transporters (c, f, i; yellow). All images are merged with DAPI showing nuclei in blue. Arrowheads indicate cells stained with either GOAT or GLUT2, SGLT1 or SGLT2, and solid arrows show cells that colocalize both GOAT and each of the glucose transporters. A magnified image of representative cells positive to GOAT and/or the glucose transporters is shown in square inset in each figure. Scale bars (µm) are indicated in each image. c, connective tissue (lamina propia + submucosa); m, mucosa.

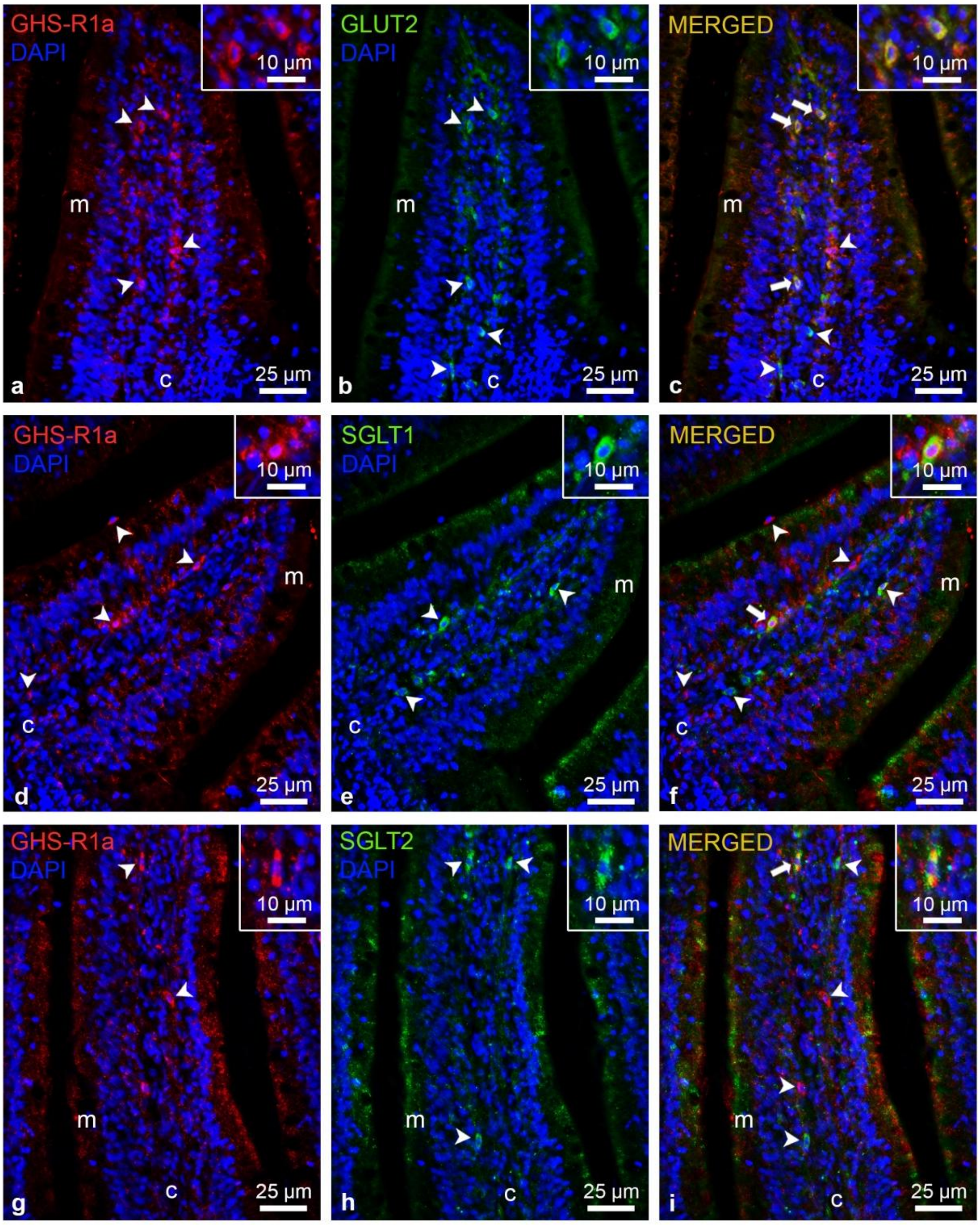

Supplementary Figure S3. GHS-R1a-like and GLUT2-like, SGLT1-like and SGLT2-like immunoreactivity in goldfish intestine detected by immunohistochemistry and assessed by fluorescence miscroscope. Transversal representative sections of intestine showing GHS-R1a-like (a, d, g; red) and GLUT2-like (b; green), SGLT1-like (e; green) or SGLT2-like (h; green) immunoreactivity, and merged images of GHS-R1a and each of the glucose transporters (c, f, i; yellow). All images are merged with DAPI showing nuclei in blue. Arrowheads indicate cells stained with either GHS-R1a or GLUT2, SGLT1 or SGLT2, and solid arrows show cells that colocalize both GHS-R1a and each of the glucose transporters. A magnified image of representative cells positive to GHS-R1a and/or the glucose transporters is shown in square inset in each figure. Scale bars (µm) are indicated in each image. c, connective tissue (lamina propia + submucosa); m, mucosa.

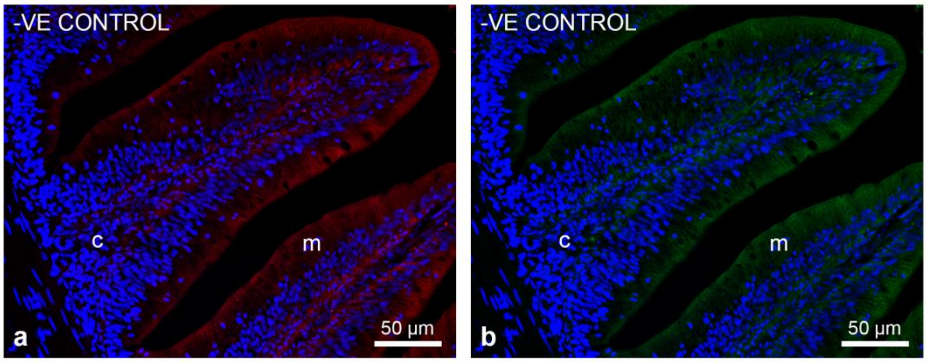

Supplementary Figure S4. Specificity of antibodies used in the present study assessed by IHC. Figure shows transversal representative sections of intestine treated only with secondary antibodies (a and b). No staining is detected in no-primary antibody negative controls. Scale bars (µm) are indicated in each image. c, connective tissue (lamina propia + submucosa); m, mucosa.

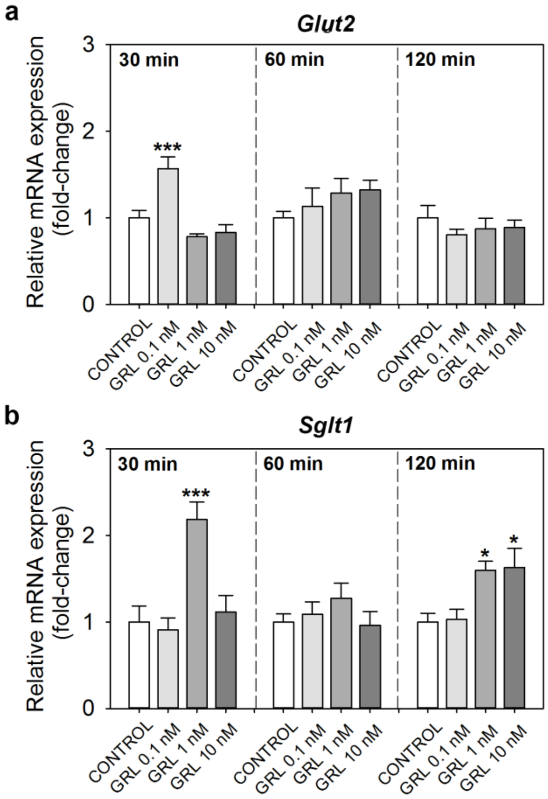

Supplementary Figure S5. Concentration and time-dependent effects of *in vitro* treatment with ghrelin (GRL) on the mRNA expression of *glut2* (a) and *sglt1* (b) in goldfish cultured liver. Cultured liver was incubated with DMEM alone (control) or containing different concentrations of ghrelin (0.1, 1 and 10 nM) during 30, 60 and 120 min. Data obtained by RT-qPCR is shown as mean + SEM (n = 6 fish). Asterisks denote statistical differences between control and treated groups assessed by ANOVA and Student-Newman-Keuls post-hoc test (* $p<0.05$, *** $p<0.001$).
